# Mitochondrial Distribution and Osteocyte Mechanosensitivity

**DOI:** 10.1007/s11914-025-00918-1

**Published:** 2025-05-22

**Authors:** Jianfeng Jin, Peter A. Nolte

**Affiliations:** 1 Department of Oral Cell Biology, Academic Centre for Dentistry Amsterdam (ACTA), University of Amsterdam and Vrije Universiteit Amsterdam, Amsterdam Movement Sciences, Gustav Mahlerlaan 3004, Amsterdam, 1081 LA The Netherlands; 2 Department of Orthopedic Surgery, Spaarne Gasthuis, Spaarnepoort 1, 2134 TM Hoofddorp, The Netherlands

**Keywords:** Cell communication, Mechanical loading, Mechanotransduction, Mitochondria, Mitochondrial distribution, Osteocytes

## Abstract

**Purpose of Review:**

Mechanical loading of bone is an important physical stimulus for bone tissue remodeling and adaptation. It is transmitted from the extracellular matrix all the way to the osteocyte nucleus via the extracellular matrix-integrin-cytoskeleton-nucleus system. Mitochondria are integral in sensing of mechanical loads to allow the cell to adapt to its environment. This review provides a background of mitochondrial distribution in osteocytes especially during mechanical loading, discussing the importance of mitochondrial distribution in osteocyte mechanosensitivity and mechanotransduction.

**Recent Findings:**

Mitochondria throughout the osteocyte are highly dynamic and provide essential metabolic and signal functions to regulate osteocyte morphology and function. They undergo the processes of fission and fusion accompanied by mitochondrial DNA distribution. The mitochondrial network structure and function in osteocytes can be regulated by mechanical loading. Interestingly, mitochondria can be transmitted by osteocytes into adjacent cells to communicate with them via tunneling nanotubes, migrasomes, and blebbisomes, causing changes in cell morphology and/or function.

**Summary:**

Mitochondrial distribution in or out osteocytes can be rearranged by physical and (bio)chemical signals via fission and fusion, as well as tunneling nanotubes, migrasomes, and blebbisomes. Mechanical loading-induced changes in mitochondria may drive signaling pathways of cell function in aging and diseases. More insights into interactions between neighbouring osteocytes and between osteocytes and other cell types would facilitate the development of new strategies to apply mitochondrial therapy for bone-related diseases.

## Introduction

Osteocytes are terminally differentiated cells derived from osteoblasts. Mature osteocytes are located with their cell bodies in the lacunae of the hard mineralized bone matrix, which makes them vulnerable to decreased supply of minerals [1]. Osteocytes are connected to one another by their long slender dendritic cell processes running through canaliculi. They communicate with other osteocytes, osteoblasts, osteoclasts, and blood vessels via their cell processes in the canaliculi of the lacuno-canalicular network. Nutrient and oxygen transport occurs by interstitial fluid flow through this lacuno-canalicular system. The osteocyte network regulates the balance between bone resorption and bone formation via communication with osteoclasts and osteoblasts [2], mainly in response to mechanical loading [3]. Although osteocyte mechanosensation and mechanotransduction are crucial processes for bone adaptation to mechanical loading and for the regulation of mineral homeostasis, little is known about how osteocytes use the lacuno-canalicular network to coordinate cellular function and communication.

Mechanosensation and the resulting mechanotransduction of biochemical signals in osteocytes is a complex but exquisite regulatory process, taking place between osteocytes and their environment, between neighboring osteocytes, and between the mechanosensors in individual osteocytes. The mechanosensors in osteocytes transmit extracellular mechanical signals into the cells and regulate mechanoresponsive gene expression. The osteocyte cytoskeleton, dendritic cell processes, integrin-based focal adhesions, connexin-based intercellular junctions, primary cilium, ion channels, and extracellular matrix are the major mechanosensors in osteocytes [4]. Mechanosensing and mechanotransduction not only happen on the cell membrane, which directly contacts external physical forces, but also inside the cell regulating deformation of the nucleus [5, 6]. However, not much is known on how the activity of organelles, e.g., the endoplasmic reticulum, golgi apparatus, lysosomes, and especially mitochondria, affect osteocyte mechanosensitivity and mechanotransduction during and/or after mechanical loading. Mitochondrial diseases (e.g. cardiovascular diseases, neurological disorders, diabetes, cancer, etc.) are the most common type of inherited metabolic disease [7, 8]. They are a group of inherited disorders characterized by defects in oxidative phosphorylation caused by mutations in nuclear and mitochondrial DNA encoding structural mitochondrial proteins or proteins involved in mitochondrial function [7]. Importantly, mitochondria (in)directly contact with various cytoskeletal filamentous networks (e.g. microtubes, intermediate filaments) which are involved in cell mechanosensitivity [9]. Therefore, mitochondria as organelles might be crucial for determining cell mechanosensitivity. Here, we discuss recent developments in the field of the function and distribution of mitochondria in the response of osteocytes to mechanical loading. We formulate some open questions to be addressed in order to obtain a better understanding of the role of mitochondria and their distribution in the processes of mechanosensing and mechanotransduction.

### Osteocytes and Alterations in Mitochondria

Mitochondrial components are abundantly present in the bone matrix, suggesting that they are transported extracellularly to orchestrate bone formation. Mitochondria and mitochondrial-derived vesicles are secreted from mature osteoblasts to promote osteogenic differentiation of osteoprogenitor cells [10]. This suggests that mitochondria and mitochondrial-derived vesicles secreted from osteocytes, which resemble mature osteoblasts, also affect osteoprogenitor cell differentiation. Induction of bone formation increases mitochondrial fragmentation, donut formation, and mitochondrial secretion via CD38/cADPR signaling [10]. Increasing mitochondrial fission and donut formation via Opa1 knockdown or Fis1 overexpression stimulates mitochondrial secretion and bone formation. Mitochondrial fusion promoter M1 enhances Opa1 expression and inhibits bone formation, while osteoblast-specific Opa1 deletion enhances bone mass [10]. The secretion of mitochondria and mitochondrial-derived vesicles results in increased bone formation in vivo. The morphology of mitochondria in mature osteoblasts seems to be adapted for extracellular secretion, and secreted mitochondria and mitochondrial-derived vesicles significantly increase bone formation [10]. This observation likely also occurs in osteocytes, as they are derived from mature osteoblasts.

Osteocyte apoptosis is caused by amongst others metabolic oxidative stress, and is crucial for starting the process of bone remodeling. Cortical osteocytes exhibit an oxidative status by using mitochondrial aerobic pathways. Depending on their exact location in the bone matrix, the osteocytes react differently to hypoxia, which is linked to the mitochondrial content [11]. There are many poorly functional mitochondria in osteocytes located near endocortical surfaces, where enhanced osteocyte apoptosis occurs, which in turn triggers bone remodeling. This suggests that regional differences in oxidative status of osteocytes determines their susceptibility to undergo apoptosis in response to stimuli that trigger bone remodeling [11].

### Osteocyte Sharing of Mitochondria and Cell Metabolism

Osteocytes share mitochondria via their dendritic cell processes [12]. The transfer of mitochondria occurs in a dynamic manner via the cell processes, and the movement of mitochondria depends on contact with the endoplasmatic reticulum [12]. Healthy osteocytes can transfer mitochondria into metabolically stressed osteocytes with non-functional mitochondria via the cell processes. This way, adenosine triphosphate (ATP) levels and oxygen consumption can be recovered, and reactive oxygen species (ROS) accumulation attenuated in the metabolically stressed osteocytes [12].

Mitochondria also play a crucial role in the energy-consuming process of vascularization in bone. Osteocytes supply mitochondria to their dendritic cell processes in a MIRO-1-dependent way followed by mitochondrial transfer to endothelial cells [13]. The ROS stress of damaged endothelial cells is relieved by the transfer of mitochondria from osteocytes, which plays an important role in the restoration of angiogenesis by endothelial cells. Furthermore, the osteocyte-derived mitochondria are crucial for blood vessel formation in cortical bone, where they modulate the endothelial cells by replacing the dysfunctional mitochondria, and activate signaling pathways [13].

### Mitochondrial Network Reshaping by External Forces

External forces applied to osteocytes can modify the mechanotransduction process. Forces applied inside (internal) or outside (external) the osteocyte may affect mitochondrial membrane tension and mitochondrial morphodynamics. The exact mechanism of external force transmission from the plasma membrane to the mitochondria is not yet known, but it is clear that mitochondria are dynamic systems which constantly respond to changes in mechanical and chemical cues. Application of external mechanical forces has been shown to increase fission fusion by intracellular or isolated mitochondria [6]. Moreover, the mitochondrial network might be differently organized dependent on the force regime applied to the osteocytes.

Changes in the physical and mechanical properties of the microenvironment, i.e., the extracellular matrix, result in metabolic changes via adhesion-mediated mechanosignaling [14]. The architectural characteristics of the extracellular matrix and the function of mitochondria have been shown to be linked [15]. This suggests that the reshaping of the mitochondrial network by mechanical loading might be involved in the process of osteocyte mechanotransduction.

### Mitochondrial DNA Distribution

Mitochondria play an important role in cell signaling pathways, e.g., cell cycle, apoptosis, β-oxidation of fatty acids, tricarboxylic acid cycle, and calcium homeostasis [16]. Unlike other organelles, mitochondria have their own DNA, called mitochondrial DNA (mtDNA), which is crucial for mitochondrial and cell function. Mitochondrial DNA, like mitochondria, is highly dynamic [17]. Mitochondrial DNA is distributed throughout the whole mitochondrial network [16, 18]. This is crucial for the protein distribution encoded by mtDNA in mitochondria [16, 18]. Therefore, mitochondrial dynamics (e.g. fusion and fission) greatly affect mtDNA distribution and maintenance [19]. Mitochondrial fusion facilitates the complementation between different mitochondria, as well as mtDNA in different mitochondria [20, 21]. Mitochondrial fission divides mtDNAs into different mitochondria, and helps mitochondria to refuse with another mitochondria forming a network [16]. The mtDNA distribution is closely linked to the mitochondrial network [16]. However, the mechanism of mtDNA distribution throughout the whole mitochondrial network is not fully understood [16]. Mitochondrial DNA is in close proximity to the Drp1-dependent mitochondrial fission site [16, 22–24]. This is highly conserved in mammalian and yeast cells [16, 23, 24]. In both mammalian and yeast cells, including osteocytes, mitochondrial division happens at the contact sites of the endoplasmic reticulum and mitochondria, where the endoplasmic reticulum wraps around the mitochondria [25, 26]. Drp1 is then recruited and assembled near the mitochondria [25, 26]. Furthermore, most endoplasmic reticulum-associated mitochondrial division occurs around the nucleus [24, 27]. After mtDNA replication, endoplasmic reticulum-associated mitochondrial fission happens between the replicated mtDNA, which is located at the tip of the newly formed mitochondria [18, 23, 24]. Localizing mtDNA to the tip of the newly formed mitochondria transmits mtDNA to the distal end of the cell and further fuses with different mitochondria to regulate mtDNA distribution [16]. This mechanism explains the homogeneously distributed mtDNA and mitochondrial distribution after mtDNA replication in cells [16]. Therefore, mtDNA distribution is important during the mitochondrial dynamics in osteocytes treated with or without mechanical loading, which might contribute to the process of bone (re)modeling.

### Mitochondria Transmitted to Neighboring Cells Via Tunneling Nanotubes

**Fig. 1:**
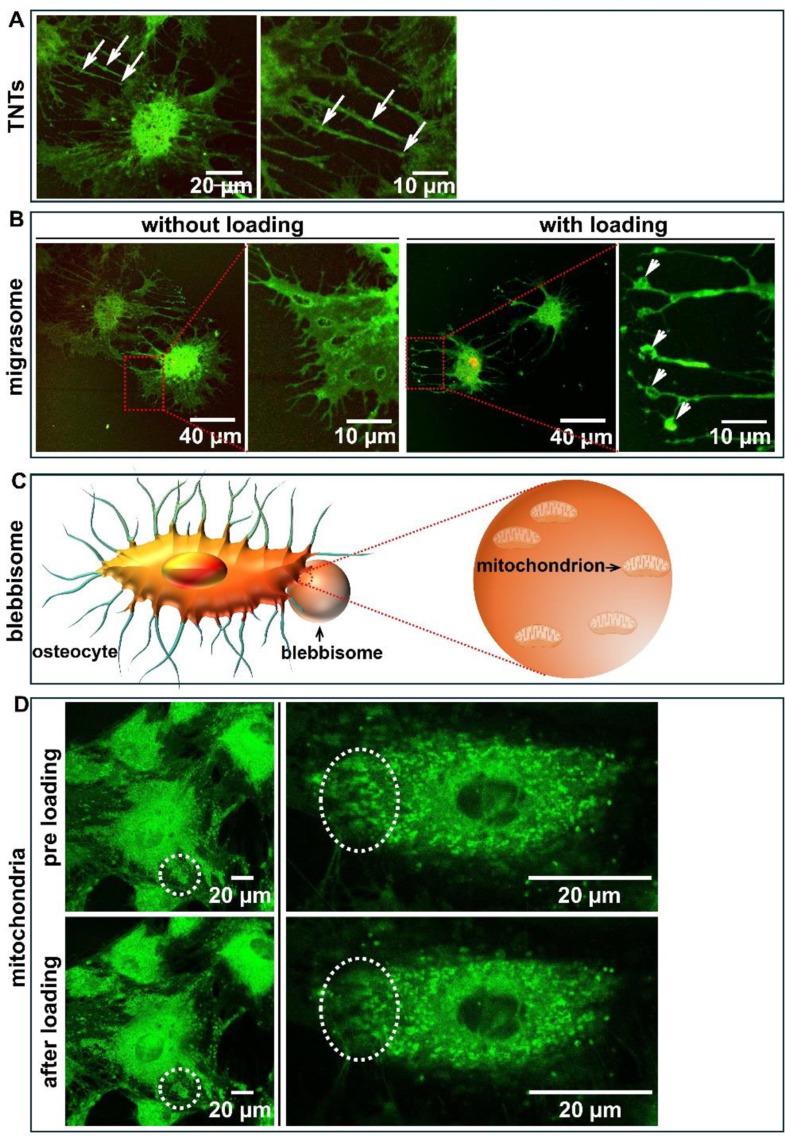
Mitochondrial transportation or distribution within a single cell or between cells without or with mechanical loading. (**A**) Mitochondrial transportation from one osteoblast to another osteoblast via tunneling nanotubes. MC3T3-E1 pre-osteoblasts treated by mechanical loading for 1 h were stained by MitoTracker for 30 min at 37 °C avoiding light. Confocal microscopy was used to observe mitochondrial distribution throughout the cell. Light green: cytoskeleton and tunneling nanotubes; bright green: mitochondria; arrows: mitochondria in tunneling nanotubes. (**B**) Mitochondrial transportation in migrasomes in osteoblasts without or with mechanical loading. Light green: cytoskeleton; bright green: mitochondria and migrasomes; arrows: mitochondria in migrasomes. (**C**) Schematic diagram of a blebbisome secreted by an osteoblast. The blebbisome contains intact healthy mitochondria. (**D**) Mitochondrial distribution in live osteoblasts pre- and post-loading Round white circle (top left): mitochondrial distribution pre-loading; round white circle (bottom left): condensed mitochondrial distribution post-loading; mitochondrial distribution (rotating and flowing, see movie at https://figshare.com/s/1edc842754030fa2c878 accessed on 19 March 2025 (doi: 10.6084/m9.figshare.28623509)) in the same cell pre- and post-loading. Round white circle (top right): more mitochondria distribution pre-loading; round white circle (bottom right): less mitochondria distribution post-loading. Green: mitochondria

Tunneling nanotubes (TNTs) are several micrometer in length and 50–200 nm in diameter without contact with the substrate [28]. They are extensions of the cell membrane, which allow the exchange of e.g., vesicles, small molecules, pathogenic components (viruses, bacteria, and pathogenic proteins), and mitochondria [29]. TNTs are positive for growth associated protein 43 (GAP43), myosin Va, myosin X, and F-actin, but mostly negative for tubulin [28–30]. However, there are significant differences in diameter, size, composition (actin or tubulin), stability (e.g., seconds, minutes, or hours), permeability (exchange of molecules or components, or cutoff of electrical signals), transport capacity (molecule transport), and regulation depending on the cell type and state [29, 31]. TNTs are formed from the cell filopodia [32]. However, TNTs and filopodia are different structures [32]. TNTs are transient structures with lifetimes ranging from minutes to hours [33]. Firstly, TNTs directly contact with other cells, while filopodia do not. Secondly, TNTs do not interact with substrates, but filopodia do. Thirdly, TNTs transport electric and small second messengers (e.g., calcium, small proteins, and microRNAs), endoplasmic reticulum/endosomal/lysosomal vesicles, and mitochondria, but filopodia do not [34, 35]. Therefore, TNTs are involved in more cell activities than filopodia. The question now arises how TNTs transfer mitochondria from one osteocyte to another osteocyte. Mitochondrial transport from mesenchymal stem cells (MSCs) to epithelial cells is regulated by Miro1 and TNFAIP2, a protein responsible for TNT formation [36]. TNFAIP2 knockdown stops mitochondrial transport [36]. Lacking Miro1 does not affect TNT formation, but mitochondrial transport through TNTs is mitigated [36]. To assess the mitochondrial network structure in bone cells in response to mechanical loading, we performed a pilot study in which we subjected MC3T3-E1 pre-osteoblasts to pulsating fluid flow (peak shear stress rate: 6.5 Pa/s, amplitude: 1.0 Pa, frequency: 1 Hz) (Fig. 1A). Although osteoblasts are less responsive to fluid flow than osteocytes, they are still sensitive to mechanical stimuli. Our pilot study using MitoTracker-stained osteoblasts, showed that two osteoblasts communicated with each other via TNTs where mitochondria were being transported (Fig. 1A). This suggests that TNTs transmitting mitochondria might also play an important role in the process of mechanotransduction in osteoyctes.

### Mitochondria Transmitted to Neighboring Cells Via Migrasomes

Migrasomes are formed on long tethers which trail behind migrating cells, called retraction fibers [37]. They undergo a growth phase during which they receive cytoplasmic contents from the cell body [37–39]. Subsequently, as the retraction fibers disassemble, they are released into the extracellular environment [37, 39]. Migrasomes can be obtained by other cells, or they may disassemble and release the contents into the extracellular environment to communicate with other cells and maintain cell homeostasis [37, 40]. Since migrasomes connect with the retraction fibers and attach to the bottom of the substrate, they might be involved in the activity of integrins, such as integrin α5β1, which is expressed in osteocytes [41], and are part of focal adhesions [42]. This α5β1 integrin has been shown to be enriched in migrasomes [42]. In bone cells, integrins are cell adhesion receptors, which regulate cell-cell and cell-extracellular matrix interaction, and act as mechanotransducers [43]. Integrins mediate mechanical signals between the cytoskeleton and the extracellular matrix, and transfer these signals into (bio)chemical/biological signals to regulate osteogenesis [43]. Our previous work showed that integrin β is involved in pre-osteoblast mechanosensitivity and may drive signaling pathways of bone cell function during mechanical loading [43]. Therefore, migrasomes might be involved in the process of mechanotransduction in osteocytes as well. Although migrasomes contain integrins, they differ from focal adhesions that are involved in the process of mechanotransduction. They have their own markers, i.e., bi-functionalheparan sulfate N-deacetylase/N-sulfotransferase 1 (NDST1), phosphatidylinositol glycan anchor biosynthesis, class K (PIGK), carboxypeptidase Q (CPQ), and EGF domain-specific O-linked N-acetylglucosaminetransferase (EOGT) [44]. Migrasomes have three main biological functions, i.e., they act as cellular information packages, as garbage disposal mechanism, and they mediate RNA and protein transfer [40]. Migrasomes dispose of damaged mitochondria when migrating cells are subjected to mitochondrial stress, called mitocytosis [45]. Mitocytosis is crucial to maintain the mitochondrial membrane potential when cells are subjected to mitochondrial stress, which is a mitochondrial quality control process [45]. Higher mitochondrial respiration in migrating cells requires more energy (or ATP) than static cells [45]. Furthermore, higher mitochondrial respiration may lead to high levels of ROS produced in migrating cells and more mitochondrial stress [45]. Importantly, migrating cells, but not static cells, generate migrasomes to mediate mitocytosis [45]. During the process of mitocytosis, the damaged mitochondria are absorbed by migrasomes [45]. To mimic the fluid shear stress induced by mechanical loading that bone cells are subjected to in vivo, we investigated the effects of fluid shear stress on mitochondrial distribution in osteoblasts (pre-osteocytes). Preliminary data showed that more migrasomes were formed on the edge of cells treated by mechanical loading for 1 h, compared to static control cells (Fig. 1B). This indicated that mechanical loading might enhance migrasome formation and mitochondrial transport via migrasomes to neighboring cells. However, little is known about migrasomes containing mitochondria in osteocytes. Future work should focus on migrasomes containing mitochondria in osteocytes with or without mechanical loading.

### Mitochondria Transmitted To Neighboring Cells Via Blebbisomes

Extracellular vesicles (EVs), containing e.g., metabolites, lipids, proteins, and nucleic acids, are crucial agents and actors in intercellular communication in cell biology with physiological or pathological conditions mediating mineralization, skeletal homeostasis, bone remodeling, and aging [46–49]. The EVs may communicate with other cells via the contact between molecules on the surface of EVs with receptors to transmit physical and/or (bio)chemical signals to escape immune surveillance and avoid degradation [46–48, 50]. The EVs are secreted into the cell surroundings by many cell types, e.g., osteocytes, bone marrow mesenchymal stem cells, osteoblasts, and osteoclasts [49]. The EVs are classified based on a variety of parameters including size. Small EVs and exosomes are generally 30–150 nm in diameter [51]. However, a new VE type has been discovered called blebbisomes which are exceptional large functional EVs (up to 20 μm) [50]. These new EVs are secreted by mouse and human cells, and have the capacity to secrete exosomes or microvesicles, or take up EVs [50]. Additionally, they move independent of cells [50]. Interestingly, blebbisomes, like cells, can undergo apoptosis, and contain intact healthy functional mitochondria [50]. These might allow them to independently execute a multitude of cell processes [50]. Since blebbisomes move independently, contain intact healthy mitochondria (Fig. 1C), and are secreted by bone cells that transfer signals and communicate with other cells, they may lead to a new research direction in bone cells including osteocytes treated with mechanical loading. Mammalian cells can release a diversity of vesicles, including blebbisomes [52]. Osteocytes are mammalian cells and highly likely form blebbisomes to regulate cell-cell communication.

### Mitochondrial Distribution in Bone Cells Treated with Mechanical Loading

Bone cells like osteocytes and osteoblasts can directly sense mechanical loading and play a crucial role in the shaping of bone [5, 53]. Osteocytes and to a lesser extent osteoblasts translate mechanical (physical) signals into (bio)chemical/biological signals via the process of mechanotransduction [5]. During mechanotransduction, osteoblasts change the shape of their body and nuclei [5]. How does mechanical loading affect the mitochondrial structure in bone cells? To answer this question, live cell imaging was performed on a MC3T3-E1 pre-osteoblast which was live-stained for the mitochondria and subjected to mechanical loading. We found that the mitochondria distributed around the nucleus. They were moving and condensed during and after mechanical loading, especially at the edge of the cell (Fig. 1D). Interestingly, mitochondria were rotating and flowing during mechanical loading (see movie at https://figshare.com/s/1edc842754030fa2c878 accessed on 19 March 2025 (doi: 10.6084/m9.figshare.28623509)). The mechanical loading-induced distribution and movement of mitochondria in osteoblasts are related to the mitochondrial structure and function, since mechanical loading did affect the mean network branch of mitochondria and *Pgc-1α* biogenetic related gene expression (unpublished data). Therefore, the distribution and movement of mitochondria in bone cells are crucial for cell function. These changes might also happen in osteoyctes since osteocytes are derived from osteoblasts, which will likely result in modulation of bone (re)modeling.

### Future Perspectives

The mitochondrial structure is located at the center of bidirectional mechanotransduction [9]. The structure responds to physical loads and undergoes changes such as fission and fusion over time by altering (bio)chemical signals that regulate the production of ATP and ROS [9]. By regulating the availability of ATP, ROS, and cytochrome c in the cytoplasm, mitochondria are able to control processes at the whole cell level and potentially even at the tissue and organ level, e.g. apoptosis, tissue contractility, and organ-level dysfunction [9]. Mitochondria contribute to a lot of diseases, as well as aging. In many diseases various mechanical factors influencing cell functions are changed [9]. The abnormal mechanical environment of the cell might be the reason that mitochondrial function becomes abnormal in these diseases [9]. During aging and in osteoporosis, the lacunar-canalicular network around the osteocytes sensing mechanical signals is altered. The mechanical signal is significantly changed in osteoporosis [54]. Osteocytes experience a higher force in osteoporotic bone than in healthy bone [54]. A higher force affects the cytoskeleton which (in)directly connects with mitochondria [55] and other organelles, e.g. endoplasmic reticulum, since mitochondria communicate with the endoplasmic reticulum via their membrane contact sites to regulate biological processes, e.g. calcium and metabolic homeostasis [56]. Based on this view, and given that many cells in the body experience mechanical perturbation, we believe that mitochondria are crucially involved in many diseases where mechanical factors are changed. Currently, the role of mitochondria in mechanobiology has not been fully unraveled, and changes in the process of mechanotransduction have not yet had an impact on medicine. Therefore, future studies should explore the connection between mitochondria and mechanobiology, which might open new strategies for the understanding and treatment of many human diseases.

## Conclusions

**Fig. 2:**
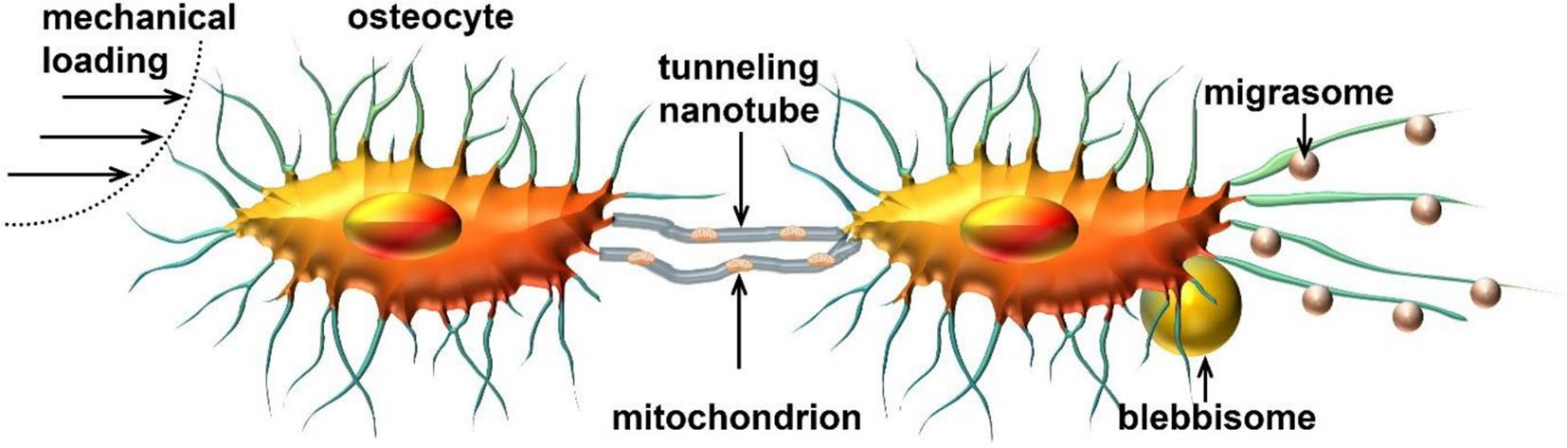
Schematic diagram of mitochondria transmitted by tunneling nanotubes, migrasomes, or blebbisomes formed by osteocytes treated by mechanical loading

Mitochondrial distribution in or out osteocytes can be rearranged by physical and (bio)chemical signals via fission and fusion, as well as tunneling nanotubes, migrasomes, and blebbisomes. The mitochondrial network structure and function are affected by mechanical loading (Fig. 2), indicating that mitochondrial distribution in osteocytes might regulate the processes of mechanosensing and mechanotransduction. The latter process of mechanotransduction is used by osteocytes to integrate information from their physical environment, and to translate the mechanical cues into biochemical signals which cause adaptive changes in bone formation by osteoblasts and bone resorption by osteoclasts. Here we reviewed the emerging relations between mechanical loading, mitochondrial distribution in osteocytes and mature osteoblasts, and bone metabolism. These relations are sometimes, but not always, well established. The reported observations raise amongst others the following questions. Are mitochondrial fission and fusion necessary for mechanosensing by osteocytes? Is increased mitochondrial fragmentation possibly a diagnostic biomarker for certain osteocyte-related diseases? Is it possible to identify a common mitochondrial mechanophenotype in osteocytes with similar mechanosensitivity, based on the fact that the mechanical properties of mitochondria determine mitochondrial distribution and dynamics? To convert mitochondrial mechanobiology in osteocytes into an efficient diagnostic and prognostic method, technical innovations and new conceptual insights are needed. More insight into the morphological, metabolic, and mechanical properties of mitochondria in osteocytes should lead to an improved understanding of mitochondria-related disease progression involving osteocytes and osteocyte-related pathologies.

## Key References

10. Liao, P.; Chen, L.; Zhou, H.; Mei, J.; Chen, Z.; Wang, B.; Feng, J.Q.; Li, G.; Tong, S.; Zhou, J.; et al. Osteocyte mitochondria regulate angiogenesis of transcortical vessels. *Nat. Commun.***2024**, *15*, 2529–2542, 10.1038/s41467-024-46095-0.
  This study shows that osteocytes regulate the homeostasis of transcortical vessels by transferring mitochondria to the endothelial cells of transcortical vessels. The results provide new insights into osteocyte-transcortical vessel interactions, and inspire the potential application of mitochondrial therapy for bone-related diseases.
34. Ma, L.; Li, Y.; Peng, J.; Wu, D.; Zhao, X.; Cui, Y.; Chen, L.; Yan, X.; Du, Y.; Yu, L. Discovery of the migrasome, an organelle mediating release of cytoplasmic contents during cell migration. *Cell Res.***2015**, *25*, 24–38, 10.1038/cr.2014.135.
  This study reports the discovery of migracytosis, a cell migration-dependent mechanism for releasing cellular contents, and migrasomes, the vesicular structures that mediate migracytosis.
40. Jin, J.; Zandieh-Doulabi, B. Low, but ot high, pulsating fluid shear stress affects matrix extracellular phosphoglycoprotein expression, mainly via integrin β subunits in pre-osteoblasts. *Curr. Issues Mol. Biol.***2024**, *46*, 12,428–12,441, 10.3390/cimb46110738.
  This study reveals that both low and high PFSS affected integrin α and β subunit expression in pre-osteoblasts, while integrin β subunit expression was more altered by low PFSS. Importantly, *Mepe* gene expression was only affected by low PFSS. These results might explain the different ways that *Mepe*-induced changes in pre-osteoblast mechanosensitivity may drive signaling pathways of bone cell function at low or high impact loading.
47. Jeppesen, D.K.; Sanchez, Z.C.; Kelley, N.M.; Hayes, J.B.; Ambroise, J.; Koory, E.N.; Krystofiak, E.; Taneja, N.; Zhang, Q.; Dungan, M.M.; et al. Blebbisomes are large, organelle-rich extracellular vesicles with cell-like properties. *Nat. Cell Biol.***2025**, *27*, 438–448, 10.1038/s41556-025-01621-0.
  This study identifies very large, organelle-containing functional extracellular vesicles that act as cell-autonomous mobile communication centres capable of integrating and responding to signals in the extracellular environment.

## Data Availability

Data availability at https://figshare.com/s/1edc842754030fa2c878 accessed on 19 March 2025 (hhttps://doi.org/10.6084/m9.figshare.28623509).
